# Morphology, genetic characterization and molecular phylogeny of the poorly known nematode parasite *Cissophyllus leytensis* Tubangui & Villaamil, 1933 (Nematoda: Ascaridida) from the Philippine sailfin lizard *Hydrosaurus pustulatus* (Eschscholtz, 1829) (Reptilia: Squamata)

**DOI:** 10.1186/s13071-022-05224-8

**Published:** 2022-04-01

**Authors:** Xue-Feng Ni, Hui-Xia Chen, Zhen Xu, Xiao-Hong Gu, Liang Li

**Affiliations:** 1 Key Laboratory of Molecular Cell Biology, Ministry of Education of the People’s Republic of China, Key Laboratory of Animal Physiology, Biochemistry and Molecular Biology of Hebei Province, College of Life Sciences, Hebei Normal University, Shijiazhuang, 050024 Hebei People’s Republic of China; 2 Medical College of Hebei University of Engineering, Handan, 056002 Hebei People’s Republic of China

**Keywords:** Parasite, Nematoda, Lizard, Ascaridida, Morphology, Genetic data, Phylogeny

## Abstract

**Background:**

The genus *Cissophyllus* (Cosmocercoidea: Kathlaniidae) is a rare group of nematodes parasitic in turtles and lizards. To date, only four species have been reported in Asia and North America. However, most of them are inadequately described. The species *Cissophyllus leytensis* has never been reported since it was originally described by Tubangui and Villaamil in 1933 from the Philippine sailfin lizard *Hydrosaurus pustulatus* (Eschscholtz, 1829) (Reptilia: Squamata). Furthermore, the systematic status of *Cissophyllus/*Cissophyllinae in the family Kathlaniidae of the superfamily Cosmocercoidea remains under debate.

**Methods:**

The detailed morphology of *C. leytensis* was studied using light microscopy (LM) and, for the first time, scanning electron microscopy (SEM), based on newly collected specimens from the type host *H. pustulatus*. Six different genetic markers, including nuclear sequences [small ribosomal subunit (18S), internal transcribed spacer (ITS) and large ribosomal subunit (28S)], plus mitochondrial genes [cytochrome c oxidase subunit 1 (*cox*1), cytochrome c oxidase subunit 2 (*cox*2) and 12S small subunit ribosomal RNA gene] of *C. leytensis* were sequenced. Additionally, in order to test the validity of the subfamily Cissophyllinae and clarify the phylogenetic relationships of *Cissophyllus* and the other genera in the family Kathlaniidae, phylogenetic analyses based on 18S + 28S and ITS sequence data were performed using maximum likelihood (ML) and Bayesian inference (BI) analyses, respectively.

**Results:**

Our observations using LM and SEM revealed some previously unreported morphological features, necessitating the redescription of this poorly known species. The presence of remarkable morphological variation in the isthmus and the position of excretory pore among different individuals was found. Molecular analysis showed no intraspecific nucleotide divergence detected in the 18S, ITS, 28S, *cox*2 and 12S regions among different individuals of *C. leytensis*, but a low level of intraspecific genetic variation was found in the *cox*1 (0.52%). Our phylogenetic results showed the representatives of the Cosmocercoidea divided into four large clades (*Cosmocerca* + *Aplectana* + *Cosmocercoides* representing the family Cosmocercidae, *Cruzia* representing the subfamily Cruzinae of Kathlaniidae, *Falcaustra* + *Cissophyllus* + *Megalobatrachonema* representing the subfamily Kathlaniinae of Kathlaniidae, and *Orientatractis* + *Rondonia* representing the family Atractidae). The genus *Cissophyllus* clustered together with the genus *Megalobatrachonema* in both the ML and BI trees using ITS sequence data, but displayed a sister relationship to the genus *Falcaustra* in the ML tree and to the genera *Falcaustra* + *Megalobatrachonema* in the BI tree using 18S + 28S sequence data.

**Conclusions:**

Molecular phylogenetic results further confirmed that the family Kathlaniidae is not a monophyletic group. The subfamily Cruziinae should be moved from the hitherto-defined family Kathlaniidae and elevated as a separate family Cruziidae. The present phylogenetic results also negated the validity of the subfamily Cissophyllinae and supported the genus *Cissophyllus* assigned in the subfamily Kathlaniinae. Molecular analysis indicated that the morphological variation in the isthmus and position of excretory pore among different individuals should be considered as intraspecific variation. Moreover, some characters important for the specific diagnosis of *C. leytensis* are reported for the first time: the number of acuminate denticles (lamellae) on each lip, the chitinized pharynx with three flabellate pharyngeal plates, the presence of single medioventral precloacal papilla and the detailed morphology of caudal papillae. The present study is only the second record of *C. leytensis*.

**Graphical Abstract:**

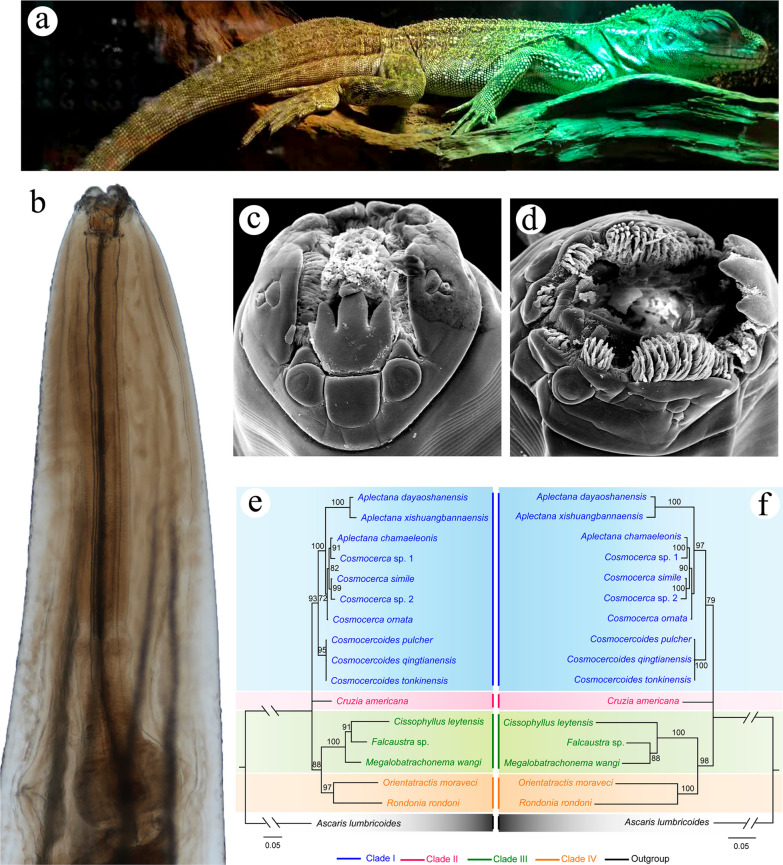

## Background

The genus *Cissophyllus* (Cosmocercoidea: Kathlaniidae) is a rare group of nematodes parasitic in turtles and lizards [1, 2]. To date, only four species have been reported in Asia and North America, including *Cissophyllus laverani* Railliet & Henry, 1912 from the Asian forest tortoise *Manouria emys* (Schlegel & Müller, 1844), the European pond turtle *Emys orbicularis* (Linnaeus, 1758) and the Malayan flat-shelled turtle *Notochelys platynota* (Gray, 1834) (Reptilia: Testudines) in India, Malaysia and Indonesia; *Cissophyllus roseus* (Leidy, 1851) from *E. orbicularis* (Linnaeus, 1758) (Reptilia: Testudines) in Indonesia; *Cissophyllus leytensis* Tubangui & Villaamil, 1933 from the Philippine sailfin lizard *Hydrosaurus pustulatus* (Eschscholtz, 1829) (Reptilia: Squamata) in the Philippines and *Cissophyllus penitus* (Leydi, 1886) from the red-eared slider turtle *Trachemys scripta elegans* (Wied-Neuwied, 1792) (Reptilia: Testudines) in the USA [2, 3]. However, most of them have been inadequately described, especially regarding the details of the cephalic structures.

The systematic status of *Cissophyllus* in the family Kathlaniidae of the superfamily Cosmocercoidea remains under debate. Railliet and Henry [4] established the genus *Cissophyllus* in 1912. In 1926, Yorke and Maplestone [5] erected the subfamily Cissophyllinae for this genus, due to the unique structure of its lips. The validity of the Cissophyllinae was accepted by Skrjabin et al. in 1964 [6] but rejected in 1978 by Chabaud [7], who placed *Cissophyllus* in the subfamily Kathlaniinae.

In the present study, the detailed morphology of *C. leytensis* was studied using light and, for the first time, scanning electron microscopy (SEM), based on newly collected specimens from the Philippine sailfin lizard *H. pustulatus*. The molecular characterization of nuclear sequences [small ribosomal subunit (18S), internal transcribed spacer (ITS) and large ribosomal subunit (28S)], plus mitochondrial genes [cytochrome c oxidase subunit 1 (*cox*1), cytochrome c oxidase subunit 2 (*cox*2) and 12S small subunit ribosomal RNA gene] of *C. leytensis* are provided for the first time. Additionally, in order to test the validity of the subfamily Cissophyllinae and clarify the phylogenetic relationships of *Cissophyllus* and the other genera in the family Kathlaniidae, phylogenetic analyses were performed based on 18S + 28S and ITS sequence data using maximum likelihood (ML) and Bayesian inference (BI) analyses, respectively.

## Methods

### Parasite collection

Nematode parasites were collected from a Philippine sailfin lizard *H. pustulatus* during a regular anthelmintic treatment by the veterinary surgeon in a zoo in Tangshan, Hebei Province, China. Specimens were washed in physiological saline and then fixed and stored in 75% ethanol, after which they were sent to the corresponding author’s lab for species identification.

### Morphological observations

For LM studies, nematodes were placed in temporary mounts and cleared in lactophenol. Photomicrographs were recorded using a Nikon^®^ digital camera coupled to a Nikon^®^ optical microscope (Nikon ECLIPSE Ni-U, Nikon Corporation, Tokyo, Japan). For SEM, the anterior and posterior ends of specimens were re-fixed in a 4% formaldehyde solution, post-fixed in 1% OsO_4_, dehydrated via an ethanol series and acetone, and then critical-point-dried. Samples were coated with gold and examined using a Hitachi S-4800 scanning electron microscope at an accelerating voltage of 20 kV. Measurements (the range, followed by the mean in parentheses) are given in micrometers (μm) unless otherwise stated. Voucher specimens were deposited in the College of Life Sciences, Hebei Normal University, Hebei Province, China.

### Molecular procedures

The midbody of one male (isthmus slightly inflated and excretory pore more or less at anterior edge of isthmus) and two females (one individual with isthmus slightly inflated and excretory pore at level of esophageal bulb, one individual with isthmus nearly as wide as corpus and excretory pore at level of esophageal bulb) were chosen for molecular analysis. Genomic DNA was extracted from each sample using a Column Genomic DNA Isolation Kit (Shanghai Sangon, China) according to the manufacturer’s instructions. DNA was eluted in elution buffer and kept at −20 °C until use. For amplifying these target sequences, the following published primers were used: the near-complete 18S ribosomal DNA (rDNA) by the primers 18SF and 18SR [8], the partial ITS region by the primers A and B [9], the partial 28S rDNA by the primers 28SF and 28SR [10], the partial *cox*1 by the primers CO1F and CO1R [11], the partial *cox*2 by the primers CO2F and CO2R [12], and the partial 12S by the primers 12SF and 12SR [13]. The cycling conditions were as described previously [14]. Polymerase chain reaction (PCR) products were checked on GoldView-stained 1.5% agarose gels and purified with the Column PCR Product Purification Kit (Shanghai Sangon, China). Sequencing of each sample was carried out for both strands. The DNA sequences obtained herein were compared (using the BLASTn algorithm) with those available in the National Center for Biotechnology Information (NCBI) database (http://www.ncbi.nlm.nih.gov). Sequences of *C. leytensis* obtained herein were deposited in the GenBank database (http://www.ncbi.nlm.nih.gov, accession numbers 18S: OM414722, OM414723; 28S: OM414718, OM414719; ITS: OM414724–OM414726; *cox*1: OM416530, OM416531; *cox*2: OM436778, OM436779, 12S: OM414720, OM414721).

### Phylogenetic analyses

Phylogenetic trees were constructed based on the 18S + 28S and ITS sequence data using ML inference with IQ-TREE and BI with MrBayes 3.2., respectively. *Ascaris lumbricoides* Linnaeus, 1758 (Ascaridida: Ascaridoidea) was chosen as the out-group. The in-group comprises 22 cosmocercoid species representing all three families in the superfamily Cosmocercoidea according to the current classifications [7, 15], including Cosmocercidae, Atractidae and Kathlaniidae. The detailed information of nematode species included in the phylogenetic analyses is provided in Table 1. Sequences were aligned using ClustalW2. We used a built-in function in IQ-TREE to select a best-fitting substitution model for the sequences according to the Bayesian information criterion [16]. The TIM3e + G4 model and the TVMe + I + G4 model were identified as the optimal nucleotide substitution model for 18S + 28S and ITS sequence data, respectively. Reliability for the ML tree was tested using 1000 bootstrap replications, and the BI tree was tested using 50 million generations. The bootstrap values over 70% are shown in the phylogenetic trees.

**Table 1:** Detailed information of representatives of Cosmocercoidea used for phylogenetic analyses

Species	Host	Locality	GenBank ID			References
			18S	ITS	28S	
*Aplectana dayaoshanensis* Chen, Ni, Gu & Li, 2021	*Hylarana spinulosa* (Smith, 1923)	China	OK045516	OK045524	OK045530	Chen et al. [22]
*Aplectana chamaeleonis* (Baylis, 1929)	*Hyperolius kivuensis* Ahl, 1931	Germany	OK045518	OK045527	OK045533	Chen et al. [22]
*Aplectana xishuangbannaensis* Chen, Gu, Ni & Li, 2021	*Polypedates megacephalus* Hallowell, 1861	China	MW329041	MW329035	MW329038	Chen et al. [23]
*Cissophyllus leytensis* Tubangui & Villaamil, 1933	*Hydrosaurus pustulatus* (Eschscholtz, 1829)	China	OM414722	OM414724	OM414718	Present study
*Cosmocerca japonica* Yamaguti 1938	*Rhacophorus arboreus* (Okada & Kawano, 1924)	Japan	–	LC052772	–	Sato et al. [25]
*Cosmocerca ornata* (Dujardin, 1845)	*Hylarana spinulosa* (Smith, 1923)	China	MW326676	MT108302	MW326675	Chen et al. [23]
*Cosmocerca simile* Chen, Zhang, Feng & Li, 2020	*Bufo gargarizans* Cantor, 1852	China	MN839758	MN839761	MN833301	Chen et al. [20]
*Cosmocerca* sp. 1	*Hoplobatrachus chinensis* (Osbeck, 1865)	China	MW329987	OK489801	MW329989	Chen et al. [23]
*Cosmocerca* sp. 2	*Bufo melanostictus* Schneider, 1799	China	MW329990	MT108303	MW329988	Chen et al. [20, 23]
*Cosmocercoides pulcher* Wilkie, 1930	*Bufo japonicus formosus*	Japan	LC018444	MH178314	LC018444	Tran et al. [17]
*Cosmocercoides qingtianensis* Chen, Zhang, Nakao & Li, 2018	*Bufo gargarizans* Cantor, 1852	China	MH178321	MH178311	MW325956	Chen et al. [18, 23]
*Cosmocercoides tonkinensis* Tran, Sato & Luc, 2015	*Acanthosaura lepidogaster* (Cuvier, 1829)	Vietnam	AB908160	AB908160	AB908160	Tran et al. [17]
*Cosmocercoides wuyiensis* Liu, Yu, Shu, Zaho, Fang & Wu, 2019	*Amolops wuyiensis* (Liu & Hu, 1975)	China		MK110871	–	Liu et al. [26]
*Cruzia americana* Maplestone, 1930	*Didelphis virginiana* Kerr, 1792	USA	U94371	–	U94757	Nadler and Hudspeth [10]
*Cruzia* sp.	*Salvator merianae* Duméril & Bibron, 1839	Brazil		MT809125	–	Unpublished
*Falcaustra sinensis* Liu, Zhang & Zhang, 2011	*Centrochelys sulcata* (Miller, 1779)	China	–	MF061681		Li et al. [14]
*Falcaustra* sp.	*Lithobates catesbeianus* (Shaw, 1802); *Indotestudo elongata* (Blyth, 1854)	Japan; China	AB818380	–	MF094270	Hasegawa et al. [27]; Li et al. [14]
*Megalobatrachonema hainanensis* Chen, Zhang & Li, 2019	*Amolops hainanensis* (Boulenger, 1900)	China	–	MH545567	–	Chen et al. [19]
*Megalobatrachonema terdentatum* (Linstow, 1898)	*Lissotriton vulgaris* (Linnaeus, 1758)	Germany	–	MN444703	–	Sinsch et al. [28]
*Megalobatrachonema wangi* Chen, Zhang, Sinsch, Scheid, Balczun & Li, 2020	*Quasipaa exilispinosa* (Liu & Hu, 1975)	China	MW325957	MH245657	MN245660	Chen et al. [21, 23]
*Orientatractis moraveci* Cavalcante, Silva, Santos, Chagas-Moutinho & Santos, 2016	*Pimelodus blochii* Valenciennes, 1840	Brazil	KX524513	–	KX524514	Cavalcante et al. [29]
*Rondonia rondoni* Travassos, 1920	*Pterodoras granulosus* (Doradidae); *Pimelodus blochii* Valenciennes	Peru; Brazil	DQ442679	–	KX524512	Wijova et al. [30]; Cavalcante et al. [29]
*Ascaris lumbricoides* Linnaeus, 1758	*Homo sapiens* Linnaeus, 1758	USA	M74585	LC422643	U94751	Müller et al. [31]; Nadler and Hudspeth [10]; Sato et al. [32]

## Results

### Morphology of *Cissophyllus leytensis* Tubangui & Villaamil, 1933 (Figs. 1, 2, 3, 4, Table 2)

**Fig. 1:**
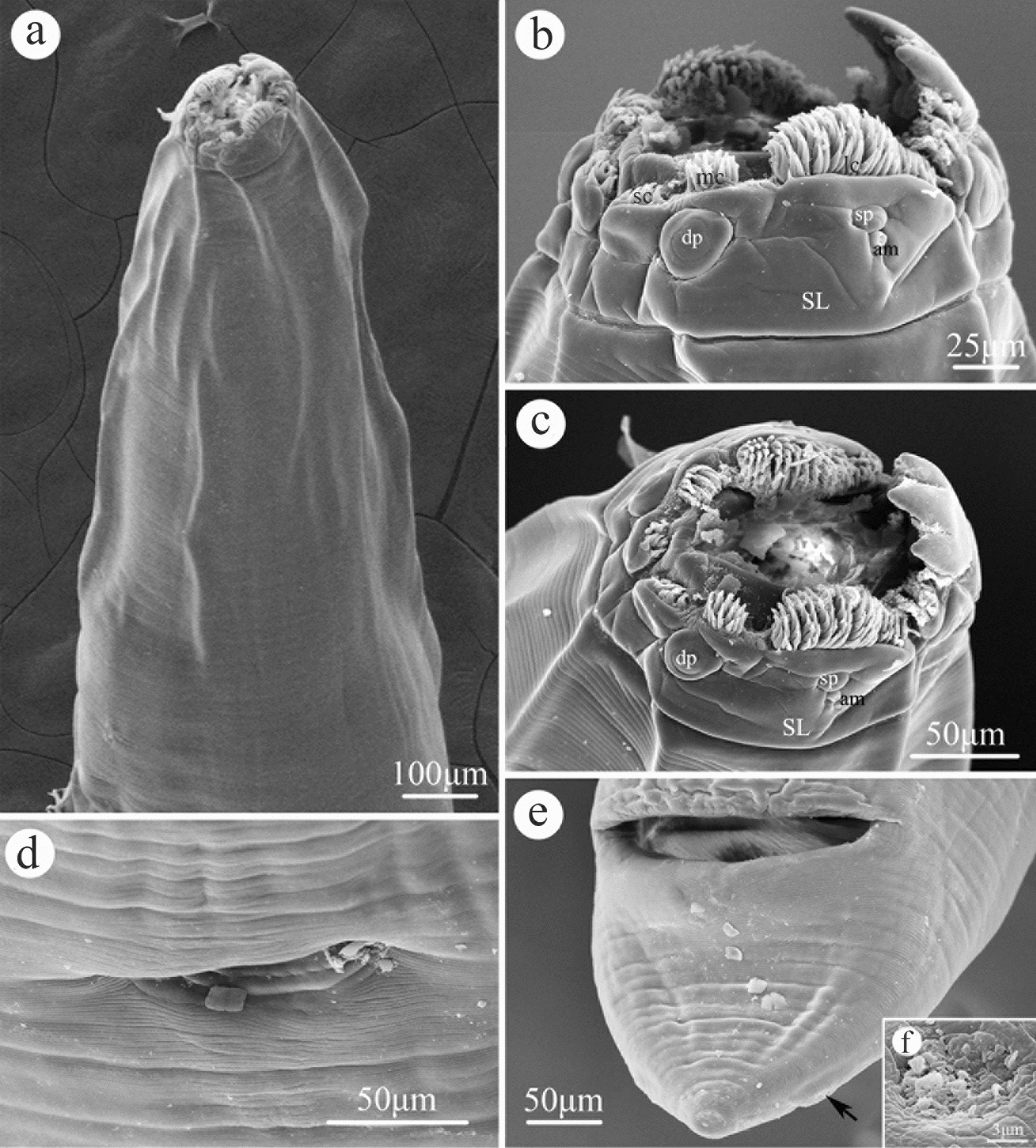
Scanning electron micrographs of female *Cissophyllus leytensis* collected from *Hydrosaurus pustulatus* (Eschscholtz, 1829) (Reptilia: Squamata) in China: **a** anterior part of body, ventrolateral view; **b** magnified image of cephalic end, lateral view; **c** cephalic end, apical view; **d** magnified image of vulva; **e** tail (black arrow showing phasmid), ventral view; **f** magnified image of phasmid. *am* amphid, *dp* large double papillae, *lc* largest cluster of acuminate denticles (lamellae), *mc* medium cluster of acuminate denticles (lamellae), *sc* smallest cluster of acuminate denticles (lamellae), *sl* subventral lip, *sp* small papilla

**Fig. 2:**
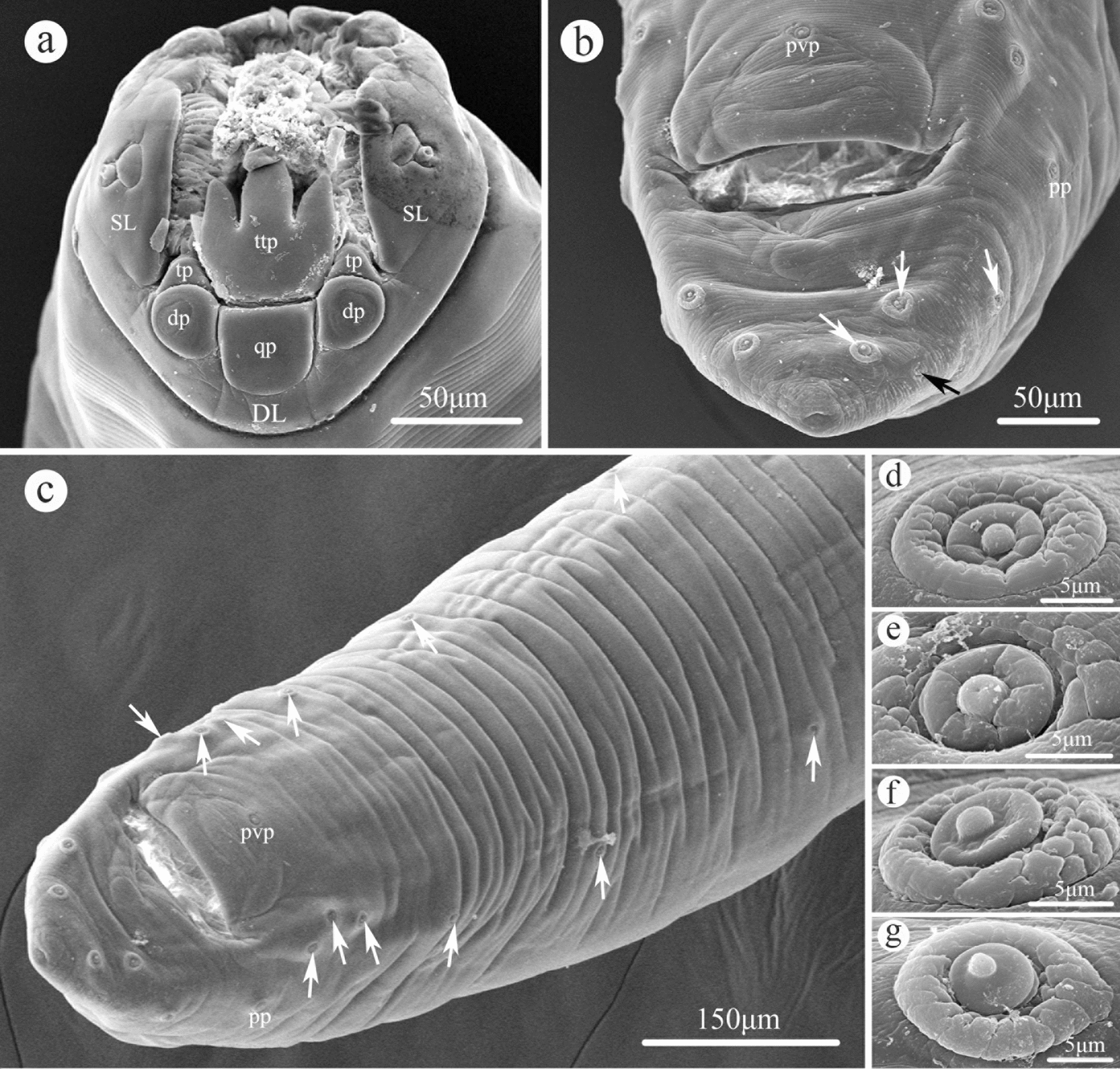
Scanning electron micrographs of male *Cissophyllus leytensis* collected from *Hydrosaurus pustulatus* (Eschscholtz, 1829) (Reptilia: Squamata) in China: **a** cephalic end, apical view; **b** tail (white arrows showing postcloacal papillae, black arrow showing phasmid), ventral view; **c** posterior part of body (white arrows showing precloacal papillae), ventral view; **d** magnified image of precloacal papilla; **e** magnified image of precloacal medioventral papilla; **f** magnified image of paracloacal papilla; **g** magnified image of postcloacal papilla. *DL* dorsal lip, *dp* large double papillae, *pp* paracloacal papilla, *pvp* precloacal medioventral papilla, *qp* single quadrate cuticular plate, *SL* subventral lip, *tp* small triangular cuticular projection, *ttp* large trilobed tooth plate

**Fig. 3:**
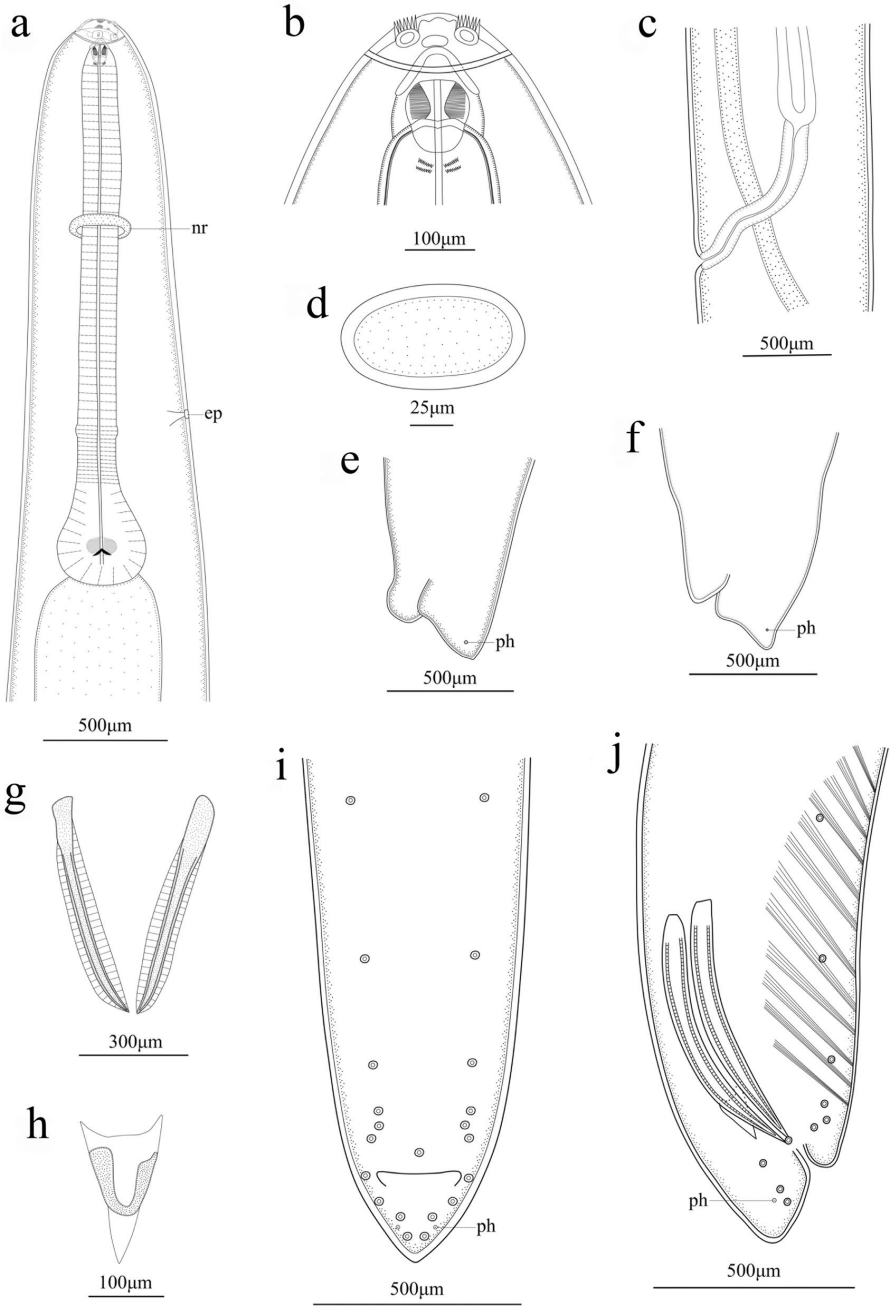
*Cissophyllus leytensis* collected from *Hydrosaurus pustulatus* (Eschscholtz, 1829) (Reptilia: Squamata) in China: **a** anterior part of female, lateral view; **b** magnified image of cephalic end, dorsal view; **c** region of vulva, lateral view; **d** egg; **e**, **f** tail of female, lateral view; **g** spicules, ventral view; **h** gubernaculum, ventral view; **i** posterior end of male, ventral view; **j** posterior end of male, lateral view. *ep* excretory pore, *nr* nerve ring, *ph* phasmid

**Fig. 4:**
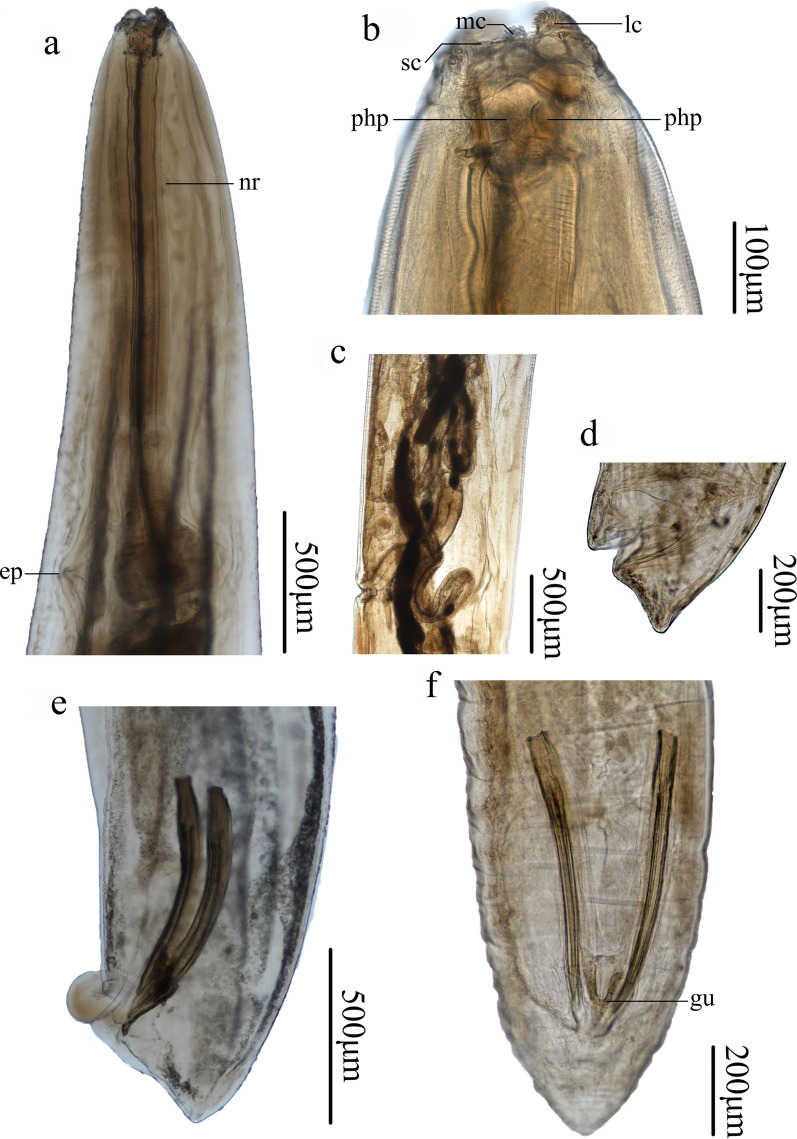
Photomicrographs of *Cissophyllus leytensis* collected from *Hydrosaurus pustulatus* (Eschscholtz, 1829) (Reptilia: Squamata) in China: **a** anterior part of male, lateral view; **b** magnified image of cephalic end, lateral view; **c** region of vulva, lateral view; **d** posterior end of female, lateral view; **e** posterior end of male, lateral view; **f** posterior end of male, ventral view. *ep* excretory pore, *gu* gubernaculum, *lc* largest cluster of acuminate denticles (lamellae), *mc* medium cluster of acuminate denticles (lamellae), *nr* nerve ring, *php* pharyngeal plates, *sc* smallest cluster of acuminate denticles (lamellae)

General. Medium-sized, whitish nematodes. Body cylindrical, maximum width at about region of middle body. Cuticle with fine transverse striations. Lateral alae absent. Oral aperture dorsoventrally elongate, surrounded by three small chitinized lips (Figs. 1a, c, 2a). Dorsal lip with one pair of large double papillae, one pair of small triangular cuticular projections (inner ridge armed with 3–5 acuminate denticles (lamellae), single quadrate cuticular plate and large trilobed tooth plate (Figs. 2a, 3b). Subventral lips each with single large double papillae, small papilla and amphid; inner ridge of each subventral lip armed with three clusters of acuminate denticles (lamellae) (smallest cluster with 6–9 denticles, largest cluster with about 80 denticles, medium one with 12–15 denticles) (Figs. 1b, c, 2a). Esophagus divided into short chitinized pharynx with three flabellate pharyngeal plates (Figs. 3a, b, 4a, b), cylindrical corpus, slightly inflated isthmus (Figs. 3a, 4a) (isthmus also nearly as wide as corpus in some specimens) and ovoid posterior bulb with valves (Figs. 3a, 4a). Nerve-ring situated at about 1/3 of total esophageal length. Position of excretory pore varied from anterior edge of isthmus to level of middle of esophageal bulb (Figs. 3a, 4a). Deirids not observed. Tail of both sexes conical, with blunt tip (Figs. 1e, 2b, c, 3e, f, i, j, 4d–f).

*Male (based on 10 specimens)*. Body 14.0–18.0 (16.6) mm long, maximum width 976–1220 (1068). Esophagus 2.00–2.39 (2.13) mm in total length, representing 11.1–17.1 (13.0) % of body length; pharynx + corpus + isthmus 1.70–2.04 (1.83) mm long, size of bulb 250–350 (305) × 260–400 (357). Nerve ring 522–807 (672) and excretory pore 1.82–2.00 (1.94) mm from anterior extremity, respectively. Posterior end of body slightly curved ventrally. Precloacal sucker absent. Spicules alate, equal in length, 600–900 (749) long, distal end sub-pointed, representing 3.89–5.63 (4.55) % of body length (Figs. 3g, j, 4e, f). Gubernaculum present, triangular, 149–248 (206) long (Figs. 3h, 4f). Caudal papillae 10 pairs in total, distributed as: six pairs of precloacal papillae (last three pairs close to each other), one pair of paracloacal papillae and three pairs of postcloacal papillae (one pair ventrolateral, two pairs ventral) (Figs. 2b–g, 3i, j). Single median ventral papilla present (Figs. 2b, c, 3i). Tail 150–260 (228) long, with rounded tip (Figs. 2b, c, 3i, j, 4e, f). Phasmids present, between last two pairs postcloacal papillae (Figs. 2b, 3i, j).

*Female (based on five specimens)*. Body 14.0–18.0 (17.0) mm long, maximum width 976–1293 (1112). Esophagus 2.15–2.49 (2.28) mm in total length, representing 12.1–15.3 (13.5) % of body length; pharynx + corpus + isthmus 1.78–2.07 (1.92) mm long, size of bulb 293–415 (361) × 341–463 (390). Nerve ring 604–894 (753) and excretory pore 1.70–1.96 (1.86) mm from anterior extremity, respectively. Vulva transverse slit, 9.40–12.3 (11.1) mm from anterior extremity, representing 63.0–68.2 (65.3) % of body length (Figs. 1d, 3c, 4c). Vagina muscular (Figs. 3c, 4c); egg oval, with smooth surface, 97–111 (105) × 53–63 (57) (*n* = 20) (Fig. 3d). Tail 250–350 (296) long, with rounded or more or less finger-like tip (Figs. 1e, 3e, f, 4d). Phasmids present (Figs. 1e, f, 3e, f).

### Taxonomic summary

#### Host and locality

Philippine sailfin lizard *H. pustulatus* (Eschscholtz, 1829) (Reptilia: Squamata) in a zoo in Tangshan, Hebei Province, China.

#### Level of infection

Single lizard infected with 15 nematodes.

#### Voucher specimen deposition

Ten males (HBNU–N-2021R0013L), five females (HBNU–N-2021R0014L), College of Life Sciences, Hebei Normal University, Hebei Province, China.

### Genetic characterization

#### Partial 18S region

Two 18S sequences of *C. leytensis* obtained herein were both 1749 base pairs (bp) in length, with no nucleotide divergence detected. In the Kathlaniidae, the 18S sequence data are available in GenBank for *Megalobatrachonema terdentatum* (MG594352–MG594364), *Megalobatrachonema wangi* (MW325957), *Cruzia americana* (U94371), *Cruzia tentaculata* (MN873564–MN873566, MN873570), *Cruzia* sp. (MT809125–MT809126), *Falcaustra ararath* (MT160412), *Falcaustra araxiana* (KM200715), *Falcaustra catesbeianae* (AB818380), *Falcaustra* sp. (MN727387, MN727389, MN727390) and *Spectatus spectatus* (KR139827). Pairwise comparison of 18S sequences of *C. leytensis* and these 10 kathlaniid species displayed 4.77% (*F. araxiana*) to 9.61% (*C. tentaculata*) nucleotide divergence.

#### Partial ITS (ITS1–5.8S-ITS2) region

Three ITS sequences of *C. leytensis* obtained herein were all 837 bp in length, with no nucleotide divergence detected. In the Kathlaniidae, the ITS sequence data are available in GenBank for *M. terdentatum* (MN444703–MN444704), *Megalobatrachonema hainanensis* (MH545567–MH545569), *M. wangi* (MN245657–MN245659), *Falcaustra sinensis* (MF061681), *Falcaustra* sp. (MN727388, MN727391, MN727392) and *Cruzia* sp. (MT809125). Pairwise comparison of ITS sequences of *C. leytensis* and these six kathlaniid species displayed 12.1% (*Cruzia* sp.) to 34.8% (*F. sinensis*) nucleotide divergence.

#### Partial 28S region

Two 28S sequence of *C. leytensis* obtained herein were both 725 bp in length, with no nucleotide divergence detected. In the Kathlaniidae, the 28S sequence data are available in GenBank for *M. terdentatum* (MN444705–MN444706), *M. wangi* (MN245660–MN245662), *M. hainanensis* (MH545569–MH545570), *F. sinensis* (MF094270), *Falcaustra* sp. (LC605539–LC605541) and *C. americana* (U94757). Pairwise comparison of 28S sequences of *C. leytensis* and these six kathlaniid species displayed 12.5% (*C. americana*) to 20.0% (*M. hainanensis*) nucleotide divergence.

#### Partial *cox*1 region

Two *cox*1 sequences of *C. leytensis* obtained herein were both 384 bp in length, with 0.52% of nucleotide divergence detected. In the Kathlaniidae, the *cox*1 sequence data are available in GenBank for *M. terdentatum* (MN444709–MN444710), *M. wangi* (MN245668–MN245670), *F. sinensis* (MF113223), *Falcaustra* sp. (MN729570–MN729572) and *C. tentaculata* (MN842776–MN842778). Pairwise comparison of *cox*1 sequences of *C. leytensis* and these five kathlaniid species displayed 12.3% (*Falcaustra* sp.) to 53.8% (*C. tentaculata*) nucleotide divergence.

#### Partial *cox*2 region

Two *cox*2 sequences of *C. leytensis* obtained herein were both 501 bp in length, with no nucleotide divergence detected. In the Kathlaniidae, the *cox*2 sequence data are available in GenBank for *C. americana* (AF179911) and *F. sinensis* (MF120240). Pairwise comparison of *cox*2 sequences of *C. leytensis* and these two kathlaniid species displayed 16.6% (*F. sinensis*) to 22.0% (*C. americana*) nucleotide divergence.

#### Partial 12S region

Two 12S sequences of *C. leytensis* obtained herein were both 469 bp in length, with no nucleotide divergence detected. In the Kathlaniidae, the 12S sequence data are available in GenBank for *M. terdentatum* (MN444707–MN444708), *M. hainanensis* (MN245666–MN245667), *M. wangi* (MN245663–MN245665) and *F. sinensis* (MF140337). Pairwise comparison of 12S sequences of *C. leytensis* and these four kathlaniid species displayed 24.7% (*F. sinensis*) to 28.6% (*M. terdentatum*) nucleotide divergence.

### Phylogenetic analyses (Figs. 5, 6)

**Fig. 5:**
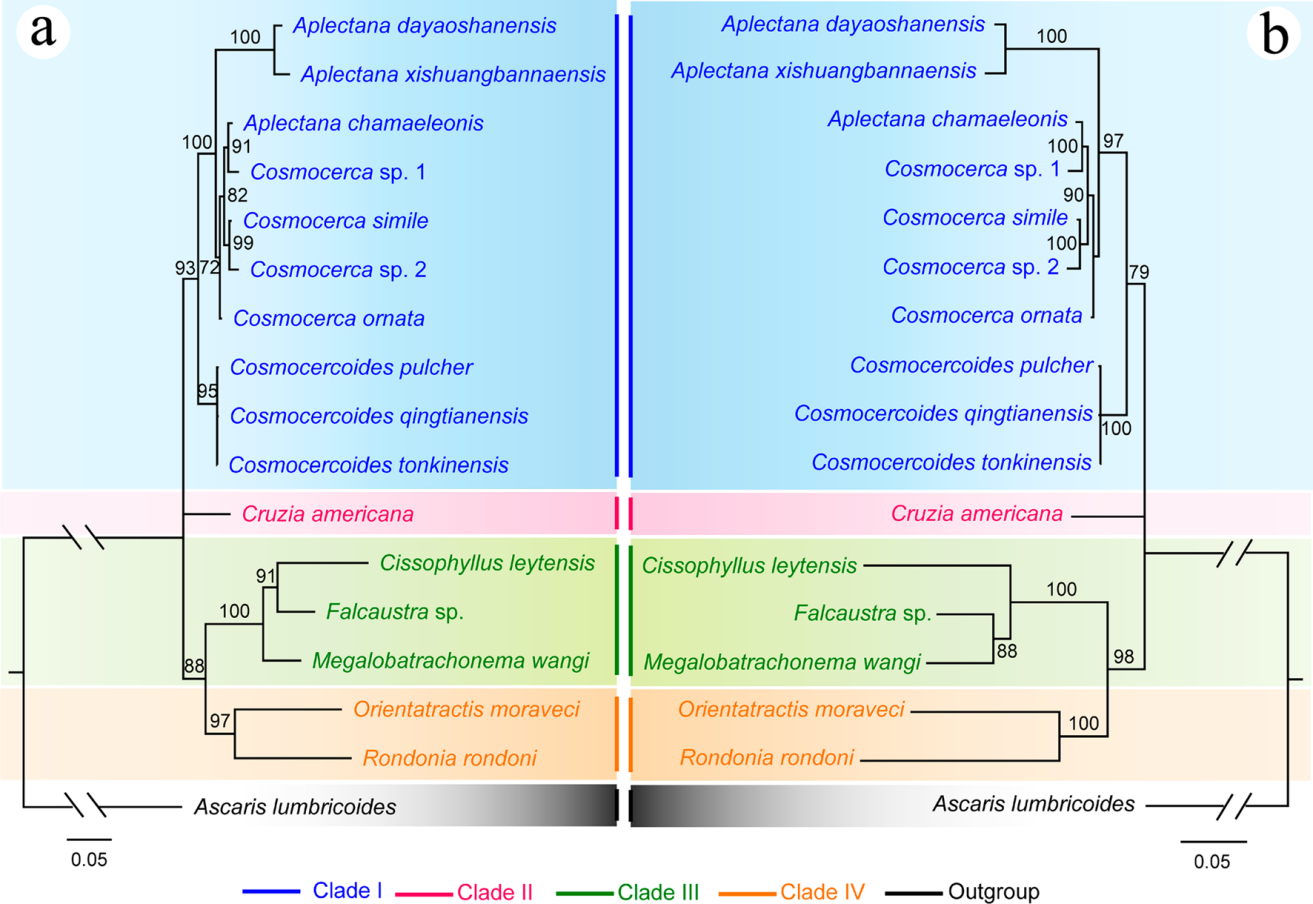
Phylogenetic relationships of representatives of the superfamily Cosmocercoidea using maximum likelihood (**a**) and Bayesian inference (**b**) analyses based on the 18S + 28S sequences. *Ascaris lumbricoides* (Ascaridida: Ascaridoidea) was chosen as the out-group. Bootstrap values exceeding 70% are shown in the phylogenetic trees

**Fig. 6:**
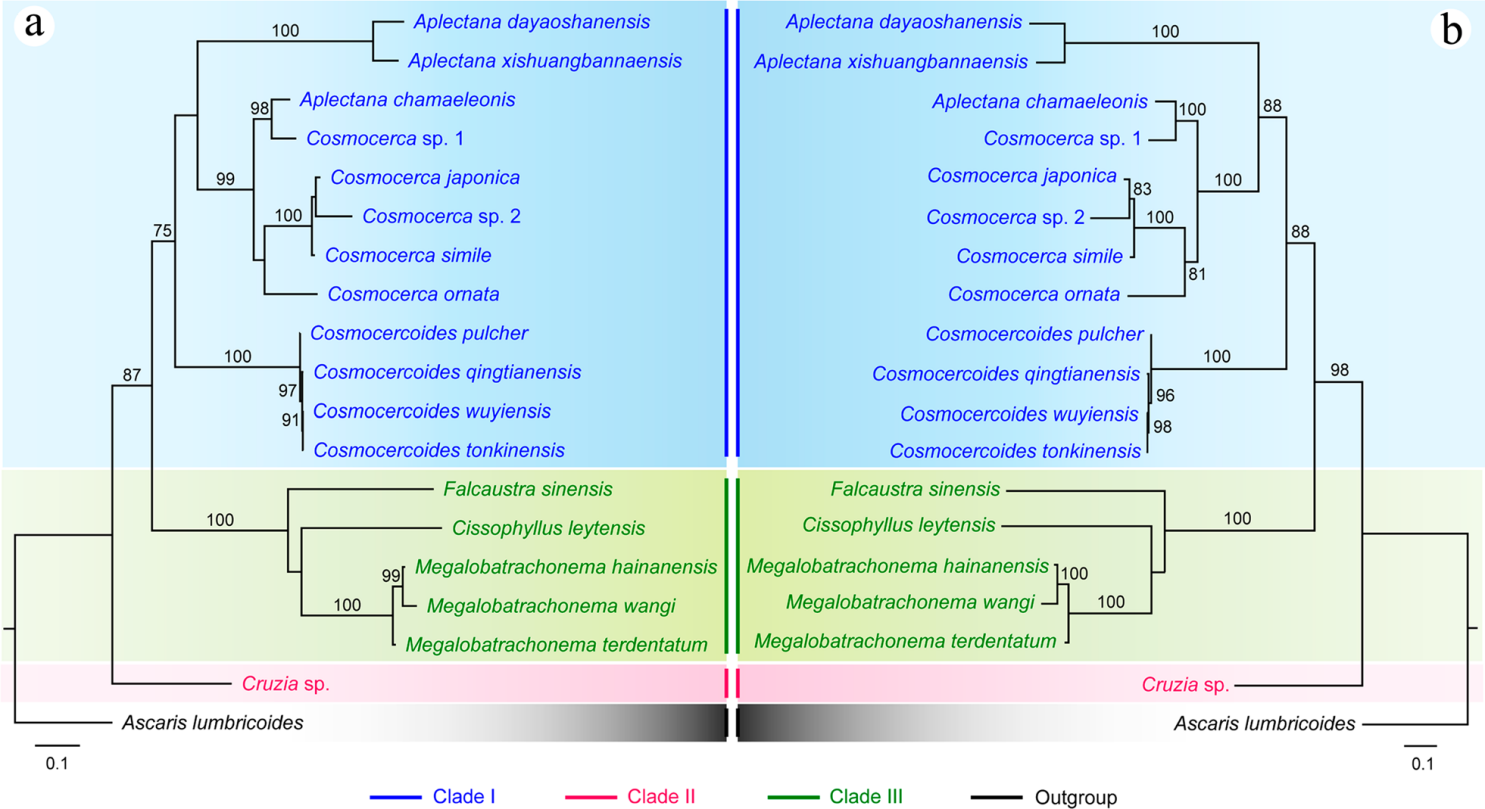
Phylogenetic relationships of representatives of the superfamily Cosmocercoidea using maximum likelihood (**a**) and Bayesian inference (**b**) analyses based on the ITS sequences. *Ascaris lumbricoides* (Ascaridida: Ascaridoidea) was chosen as the out-group. Bootstrap values exceeding 70% are shown in the phylogenetic trees

The phylogenetic results of ML and BI trees using 18S + 28S sequence data were more or less identical, with both showing the representatives of the superfamily Cosmocercoidea divided into four large clades (Fig. 5). The species of *Cosmocerca* + *Aplectana* + *Cosmocercoides* formed clade I, which represents the family Cosmocercidae. The species *C. americana* formed clade II, which represents the subfamily Cruzinae in the Kathlaniidae. The species of *Falcaustra* + *Cissophyllus* + *Megalobatrachonema* formed clade III, which represents the subfamily Kathlaniinae in the Kathlaniidae. The species of *Orientatractis* + *Rondonia* formed clade IV, which represents the family Atractidae. *Cissophyllus* and *Falcaustra* formed a sister group in the ML tree in clade III, but *Cissophyllus* clustered together with *Falcaustra* + *Megalobatrachonema* in the BI tree (Fig. 5). By contrast, the phylogenetic results of ML and BI trees using ITS sequence data showed the representatives of the superfamily Cosmocercoidea divided into three large clades, due to the lack of available ITS data for atractid species (Fig. 6). The genus *Cruzia* (clade II) is at the base of the ML and BI trees, and the genus *Cissophyllus* showed a closer relationship to *Megalobatrachonema* than *Falcaustra* with weak support (Fig. 6).

## Discussion

Tubangui and Villaamil (1933) [1] described *C. leytensis* from *H. pustulatus* in the Philippines. The morphology and measurements of the present specimens are almost identical to the original description of *C. leytensis* by Tubangui and Villaamil (1933) regarding some important taxonomical features, including the morphology of the lips, the length of the male body and total esophagus, the morphology and length of tail, spicules and gubernaculum, the number and arrangement of caudal papillae, and the absence of the precloacal sucker (see Table 2 for details). It should be noted that the present specimens were collected from the type host *H. pustulatus*. Therefore, we consider our newly collected specimens to be conspecific with *C. leytensis*. However, we observed the position of the excretory pore varied from the anterior edge of the isthmus to the level of the middle of the esophageal bulb, and the isthmus slightly inflated (slightly wider than corpus) or nearly as wide as the corpus among different individuals of our specimens. Tubangui and Villaamil (1933) [1] did not mention the intraspecific morphological variation of these characters in their description. The size of eggs and the length of females in the present study are slightly smaller than those of the original description (see Table 2 for details), which were possibly affected by the age/developmental stage or infection intensity of parasites. Some characters important for the specific diagnosis of *C. leytensis* were reported for the first time: the number of acuminate denticles (lamellae) on each lip, the chitinized pharynx with three flabellate pharyngeal plates, the presence of single medioventral precloacal papilla and the detailed morphology of caudal papillae.

**Table 2:** Morphometric comparisons of *Cissophyllus leytensis* (Nematoda: Kathlaniidae) (measurements in millimeters)

Characteristics	Present study		Tubangui & Villaamil (1933)	
	Male	Female	Male	Female
Length of body	14.0‒18.0	14.0‒18.0	13.0‒21.7	17.0‒23.5
Maximal width	0.98‒1.22	0.98–1.29	0.98–1.50	1.10–1.60
Length of entire esophagus	2.00‒2.39	2.15‒2.49	1.93‒2.20	1.96‒2.30
Distance nerve ring from anterior end	0.52–0.81	0.60–0.89	0.48‒0.52	0.56–0.60
Distance excretory pore from anterior end	1.82‒2.00	1.70‒1.96	1.30‒1.50	1.60‒1.85
Spicule length	0.60‒0.90	–	0.56‒0.90	–
Gubernaculum length	0.15‒0.25	–	0.14‒0.20	–
Number and arrangement of caudal papillae (pairs)	6, 1, 3	–	5–6, 1, 3	–
Length of tail	0.15‒0.26	0.25‒0.35	0.21–0.30	0.26‒0.50
Size of eggs	–	0.097–0.11 × 0.053–0.063	–	0.11–0.13 × 0.064–0.073
Host	*Hydrosaurus pustulatus*		*Hydrosaurus pustulatus*	
Locality	China		Philippines	

In the genus *Cissophyllus*, only *C. leytensis* has been reported from a lizard, with the other three species *Cissophyllus laverani*, *C. roseus* and *C. penitus* all from turtles. *Cissophyllus leytensis* can be easily distinguished from *C. laverani*, *C. roseus* and *C. penitus* by the absence of a precloacal sucker (vs. the presence of remarkable precloacal sucker). It is very interesting that the species of *Cissophyllus* parasitic in different hosts (lizard and turtles) showed such distinct morphological differences. However, we do not think that it is reasonable to erect a new genus or subgenus for *C. leytensis*, because the other generic diagnostic characters of the four species are almost coincident. But the true phylogenetic relationships of the four species should be investigated using phylogenetic analysis based on genetic sequences in the future.

In recent years, some studies have started to expand their morphological descriptions of new species of the superfamily Cosmocercoidea with DNA sequence data [17–23]. Nevertheless, the vast majority of the c. 410 currently recognized species in the Cosmocercoidea [15] were defined under the traditional morphospecies concept. Within *Cissophyllus*, none of the four currently recognized species had been characterized using molecular markers since they were originally described. In the present study, the genetic characterization of the partial 18S, ITS, 28S ribosomal DNA, and the partial mitochondrial *cox*1, *cox*2 and 12S of *C. leytensis* are provided for the first time. Based on the molecular analysis of *C. leytensis*, low levels of intraspecific nucleotide differences were noted only in the *cox*1 region, but high levels of interspecific genetic variation in all six genetic markers was clear among the genera of Kathlaniidae. These genetic data of *C. leytensis* obtained herein will be valuable for further investigations on the species identification, population genetics and phylogeny of this poorly known group.

Our phylogenetic analyses based on 18S + 28S and ITS sequence data both showed that the family Kathlaniidae is not a monophyletic group. The present results are consistent with some recent phylogenetic studies [22, 23]. According to the classification by Chabaud (1978) [7], the Kathlaniidae includes three subfamilies, namely Kathlaniinae, Cruziinae and Oxyascaridinae. However, Chabaud’s classification has been challenged by some traditional taxonomical studies and recent molecular phylogenetic studies [22–24]. Our phylogenetic results supported the subfamily Cruziinae moved out from the hitherto-defined family Kathlaniidae and elevated to a separate family, which agreed with the proposal by Travassos (1917) and Skrjabin et al. (1960) [33, 34]. Moreover, the present phylogenetic results supported the genus *Cissophyllus* belonging to the subfamily Kathlaniinae, which is congruent with the traditional classification of Chabaud (1978) [7]. The subfamily Cissophyllinae proposed by Yorke and Maplestone (1926) and Skrjabin et al. (1976) is invalid. The highly specialized structure of the cephalic end of *Cissophyllus* species can only be treated at the level of a generic diagnostic character.

## Conclusions

Molecular phylogenetic results further confirmed that the family Kathlaniidae is not a monophyletic group. The subfamily Cruziinae should be moved from the hitherto-defined family Kathlaniidae and elevated as a separate family Cruziidae. The present phylogeny also negated the validity of the subfamily Cissophyllinae and supported the genus *Cissophyllus* assigned in the subfamily Kathlaniinae. Molecular analysis indicated that the presence of morphological variation in the isthmus and position of excretory pore among different individuals should be considered as intraspecific variation. Moreover, some characters important for the specific diagnosis of *C. leytensis* are reported for the first time: the number of acuminate denticles (lamellae) on each lip, the chitinized pharynx with three flabellate pharyngeal plates, the presence of single medioventral precloacal papilla and the detailed morphology of caudal papillae. The present study is only the second record of *C. leytensis*.

## Data Availability

The nuclear and mitochondrial DNA sequences of *Cissophyllus leytensis* obtained in this study were deposited in GenBank database. Voucher specimens of *C. leytensis* were deposited in the College of Life Sciences, Hebei Normal University, Hebei Province, under the accession numbers HBNU–N-2021R0013L, HBNU–N-2021R0014L, China.

## Abbreviations

am: Amphid
BI: Bayesian inference
*cox*1: Cytochrome c oxidase subunit 1
*cox*2: Cytochrome c oxidase subunit 2
DL: Dorsal lip
dp: Large double papillae
ep: Excretory pore
gu: Gubernaculum
ITS: Internal transcribed spacer
lc: Largest cluster of acuminate denticles (lamellae)
LM: Light microscopy
mc: Medium cluster of acuminate denticles (lamellae)
ML: Maximum likelihood
nr: Nerve ring
PCR: Polymerase chain reaction
php: Pharyngeal plates
pp: Paracloacal papilla
pvp: Precloacal medioventral papilla
qp: Single quadrate cuticular plate
sc: Smallest cluster of acuminate denticles (lamellae)
SEM: Scanning electron microscopy
SL: Subventral lip
sp: Small papilla
tp: Small triangular cuticular projection
ttp: Large trilobed tooth plate
12S: Small subunit ribosomal RNA gene
18S: Small ribosomal subunit
28S: Large ribosomal subunit
